# Manipulation of the Innate Immune Response by Varicella Zoster Virus

**DOI:** 10.3389/fimmu.2020.00001

**Published:** 2020-01-24

**Authors:** Chelsea Gerada, Tessa M. Campbell, Jarrod J. Kennedy, Brian P. McSharry, Megan Steain, Barry Slobedman, Allison Abendroth

**Affiliations:** Infectious Diseases and Immunology, Faculty of Medicine and Health, Charles Perkins Centre, University of Sydney, Sydney, NSW, Australia

**Keywords:** varicella–zoster virus, immune evasion, innate immune response, herpes zoster (HZ), varicella (chickenpox)

## Abstract

Varicella zoster virus (VZV) is the causative agent of chickenpox (varicella) and shingles (herpes zoster). VZV and other members of the herpesvirus family are distinguished by their ability to establish a latent infection, with the potential to reactivate and spread virus to other susceptible individuals. This lifelong relationship continually subjects VZV to the host immune system and as such VZV has evolved a plethora of strategies to evade and manipulate the immune response. This review will focus on our current understanding of the innate anti-viral control mechanisms faced by VZV. We will also discuss the diverse array of strategies employed by VZV to regulate these innate immune responses and highlight new knowledge on the interactions between VZV and human innate immune cells.

## Introduction

Varicella zoster virus (VZV) is a medically important human herpesvirus and infections are extremely common, with seroprevalence rates >90% in most populations around the world. Primary VZV infection causes chickenpox (varicella). The virus then establishes life-long latency in sensory neurons from where it can reactivate years later to cause shingles (herpes zoster), which is typified by a skin rash with a dermatomal distribution. Following herpes zoster rash resolution, many individuals continue to experience severe neuropathic pain, termed post-herpetic neuralgia (PHN), that can persist for months to years (1).

VZV is a member of the alphaherpesvirus family and is closely related to herpes simplex virus type 1 (HSV-1). VZV is genetically stable, a property which is demonstrated by little nucleotide variation between isolates (2). The VZV virion is composed of a double stranded (ds) deoxyribonucleic acid (DNA) genome, an icosahedral capsid, tegument, and envelope (3). The genome resides within the icosahedral capsid, which is composed of 162 capsomeres. The VZV genome is the smallest of the alphaherpesviruses and is composed of 71 unique open reading frames (ORFs) (4). Once VZV enters a host cell, a temporal cascade of gene expression occurs in which immediate early transactivating genes are expressed (5). This allows for the expression of early genes which are involved in VZV DNA replication. After viral DNA replication, late genes which encode for structural VZV proteins such as glycoproteins are expressed to allow the virus to egress from the host cell. VZV can be distinguished from other members of the alphaherpesvirus family as it exhibits a highly restricted host specificity to human and simian cells (6, 7).

One of the major obstacles in studying VZV pathogenesis and the host immune response is the virus' strict species specificity. Thus, our current knowledge has stemmed from clinical studies and examination of human tissues, experimental models of VZV infection *in vitro* utilizing human cells and infection of human tissue xenografts implanted in severe combined immunodeficient (SCID-hu) mice, as well as observations from the simian varicella virus (SVV) infection of non-human primates, which has been used to model VZV infection *in vivo* (8). In this review, we draw upon a range of such studies to provide an update on how VZV interacts and manipulates early innate anti-viral responses in cell-types critical to VZV disease, encompassing both immune and non-immune cells.

## Pathogenesis of VZV

### Pathogenesis of Primary VZV Infection

**Figure 1:**
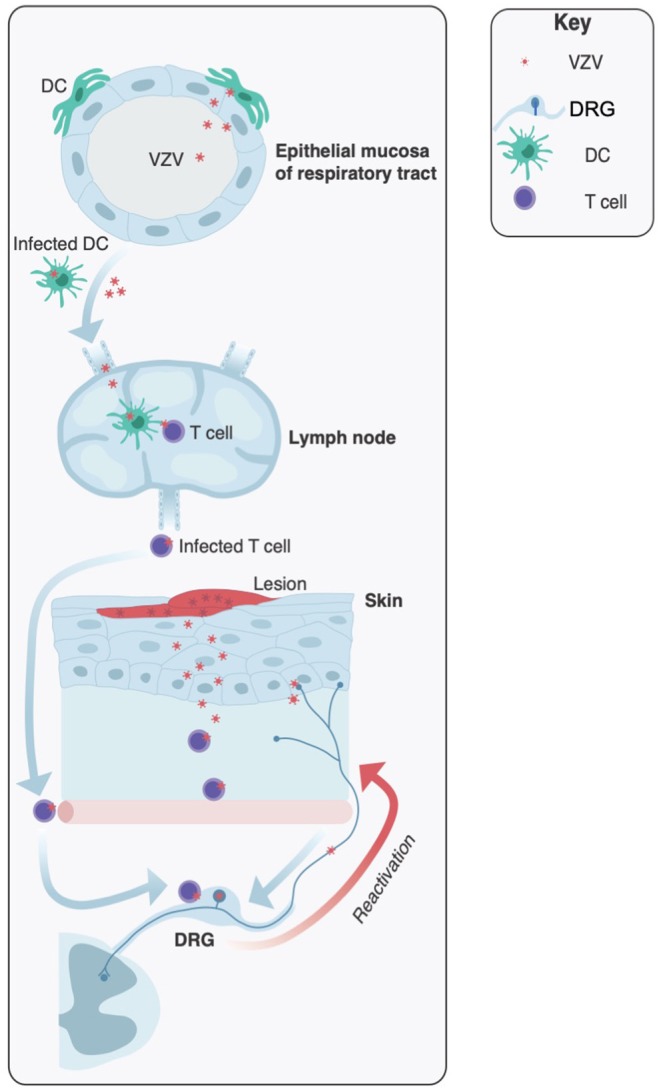
Key sites of infection during varicella zoster virus pathogenesis. Initial infection is usually mediated by inhalation of highly infectious particles from patients undergoing acute varicella infection. It is proposed that VZV initiates infections in the upper respiratory tract, infecting the epithelial mucosa. Local dendritic cells (DCs) become infected and virus is transferred to the lymph nodes (and tonsils) where T cells are infected. Viremia leads to VZV dissemination to the skin and sensory neurons of the dorsal root ganglia (DRG) where the virus establishes a latent infection. Later in life VZV has the potential to reactivate and travel via anterograde spread to the skin, resulting in productive infection and the characteristic herpes zoster rash.

In order to appreciate the innate anti-viral immune response to VZV it is important to review the pathogenesis of VZV infection (Figure 1). Primary infection is initiated through exposure to highly infectious vesicular fluid from cutaneous lesions or through inhalation of infectious respiratory droplets from an individual with varicella. It is presumed that VZV initiates infection in the epithelial mucosa of the upper respiratory tract, from where the virus gains access to immune cells in the tonsils and local lymphoid tissue. It has been postulated that dendritic cells (DCs) are the first immune cell type to become infected in the respiratory mucosa (9, 10). DCs extensively interact with other cells via direct contact, which would provide a mechanism for VZV to be transmitted to other immune cells in the tonsils, especially T cells (11). VZV infection then progresses to a viremia, which may include dissemination of virus to internal organs. During this phase of infection, there is a prolonged incubation period of typically 14–16 days in which there are no detectable symptoms. This is followed by the infection progressing back to the respiratory mucosa and spreading to the skin. It is at this site that symptoms develop, most notably via the infection of keratinocytes which results in a vesiculopustular exanthema, with highly infectious lesions, spread across the body, as well as mucous membranes such as the oral cavity (1, 12–14). During primary infection, VZV dissemination around the body is considered to be facilitated by the migration of infected T cells (15–17). This model of VZV pathogenesis is supported by clinical studies of immunocompetent patients with varicella, where VZV could be cultured from peripheral blood mononuclear cells (PBMCs) isolated during the incubation phase of disease and peaking before the onset of the vesicular cutaneous rash (18, 19).

Primary varicella is resolved by the host immune response typically within 1–2 weeks. However, in the absence of a fully functional immune response, VZV may spread to other sites including the central nervous system (CNS) and lungs. Dissemination of infection may result in a number of serious complications, including VZV encephalitis, cerebellar ataxia, demyelinating neuropathy, myelitis, and pneumonia (20, 21).

During primary infection, despite a robust immune response, VZV is not completely eliminated from the host but rather the virus gains access to neurons in the sensory ganglia and establishes a life-long latent infection (22–24). The virus spreads to the sensory ganglia through retrograde axonal transport from free nerve endings in the skin (25, 26), and potentially via hematogenous spread in immune cells infiltrating the ganglia (24, 27, 28). It has also been proposed that VZV can establish latency in the enteric nervous system, providing a possible explanation for cases linking VZV with gastrointestinal disorders (29, 30).

### Pathogenesis of VZV Reactivation and Latency

Reactivation from latency causes herpes zoster (shingles), a neurocutaneous disease which occurs in 10–20% of seropositive individuals and involves anterograde axonal transport of virus from the reactivating ganglia to the innervating dermatome (31–33). The incidence of herpes zoster is thought to correlate with a reduction in VZV-specific T cell mediated immunity (34, 35). Specifically, increasing age is a strong predisposing factor, with ~68% of herpes zoster cases occurring in individuals over 50 years of age (36). Concomitant infection with other pathogens can also influence VZV reactivation. Adults with disseminated non-tuberculous mycobacterial infections can reactivate latent VZV infection and this is associated with anti-IFNγ autoantibodies (37). Additionally, there has been evidence of concurrent reactivation of HSV-1 and VZV, however this occurs rarely (38). It is unclear whether specific pathogens can increase the likelihood of VZV reactivation or whether VZV reactivation during other infections is due to a weakened VZV specific immune response.

Herpes zoster rash development is often preceded by a prodrome of dermatomal pain and is clinically characterized by a unilateral cluster of lesions typically across a single dermatome, accompanied by localized pain of varying intensity, and neuritis. The cutaneous lesions contain infectious virus and provides another reservoir for virus transmission to other susceptible individuals (39). Occasionally VZV reactivates in individuals experiencing dermatomal restricted neuropathic pain but without cutaneous lesions present; a condition known as zoster sine herpete (pain without rash) (40).

Herpes zoster has the potential to severely impact an individual's quality of life. The most common complication of herpes zoster is PHN which is a pain persisting for months to years after herpes zoster rash resolution (41). PHN occurs in 5–30% of people who experience herpes zoster and the prevalence and severity increases dramatically with advancing age (42). To date the mechanisms underpinning PHN are not yet fully understood. Other complications associated with VZV reactivation include meningitis, vasculopathy (including giant cell arteritis), myelopathy, ocular manifestations including herpes zoster opthalmicus, acute retinal necrosis, and progressive outer retinal necrosis (24, 39).

## VZV Modulates Apoptosis in a Cell Type Specific Manner

Programmed cell death is a critical component of the intrinsic and innate immune response, as it allows for the rapid elimination of damaged or infected cells (43). Viral infection can trigger programmed cell death via multiple pathways such as sensing of the virus through pattern recognition receptors (PRR), damage to host cell DNA and endoplasmic reticulum stress (44). The main forms of programmed cell death initiated by viral infection include apoptosis, necroptosis, and pyroptosis (44). Apoptosis is a non-inflammatory form of programmed cell death, which can be distinguished by the cleavage of caspase 3 and has been considered to be the main cell death mechanism used (45). Necroptosis is an inflammatory form of cell death which shares some of the apoptosis biochemical pathway. In particular, if components of the apoptosis pathway are inhibited, necroptosis can be initiated, eventually causing the phosphorylation of mixed lineage kinase domain-like (MLKL) and the formation of pores at the cell membrane (46). Pyroptosis is mediated by the inflammasome which contains a PRR from the Nod-like receptor (NLR) family, the adaptor ASC and caspase-1. Inflammasome activation causes cell membrane disruption and is therefore also an inflammatory form of programmed cell death (46).

In the context of VZV, apoptosis has been the most comprehensively investigated programmed cell death pathway. Apoptosis contains distinct biochemical pathways, which are highly complex and involve an energy dependent cascade of molecular events (47, 48). Three apoptosis pathways have been identified: the extrinsic, intrinsic, and perforin/granzyme pathway. All of these pathways converge in the cleavage of caspase 3, the major hallmark of apoptosis induction. This causes DNA fragmentation, nuclear, and cytoskeletal protein degradation, formation of apoptotic bodies, and engulfment by phagocytes (49). Apoptosis can be triggered in viral infection through cellular damage, viral detection through PRRs or through natural killer (NK) cell or cytotoxic T lymphocyte (CTL) recognition of target cells (43). CTLs and NK cells can kill virally infected cells through the expression of FasL which binds to Fas on the target cell and induces the extrinsic apoptotic pathway or through the delivery of perforin and granzyme B (50).

### VZV Modulation of Apoptosis in Neuronal and Non-neuronal Cells

**Figure 2:**
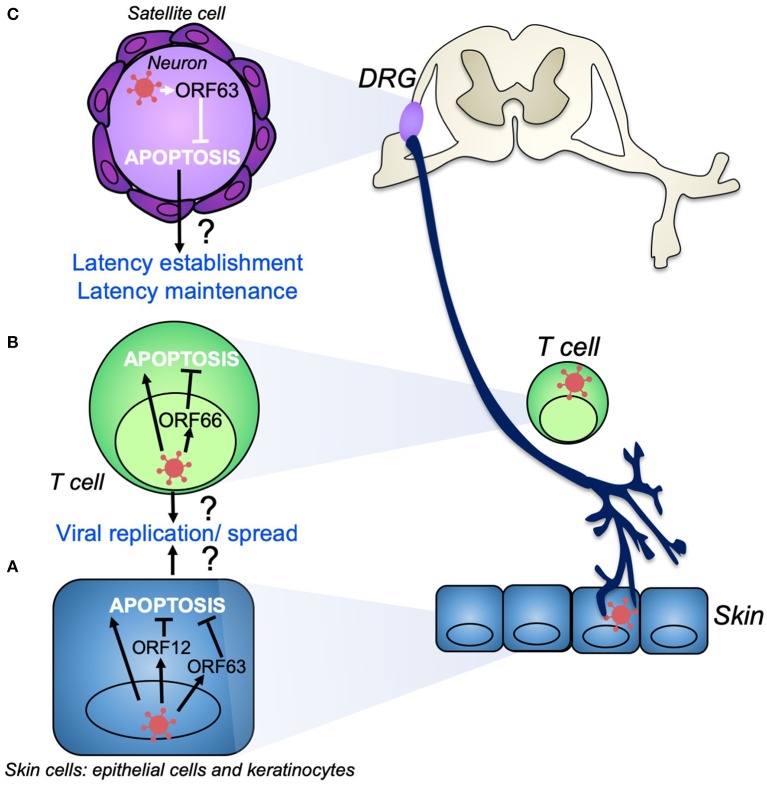
VZV modulation of apoptosis during productive infection and the establishment of latency. During productive infection VZV infects skin cells **(A)** such as keratinocytes, fibroblasts, and epithelial cells. VZV induces apoptosis in skin cell types, despite the production of anti-apoptotic gene products such as VZV ORF12 and ORF63, which may act to delay apoptosis to ensure efficient viral replication and spread. **(B)** T cells are also infected during primary infection and act as a conduit to transport VZV to the skin and dorsal root ganglia (DRG). VZV induces apoptosis in T cells as well as other immune cells. VZV ORF66 may act to delay T cell apoptosis to promote viral dissemination. VZV establishes life-long latency in sensory neurons of the DRG **(C)**. VZV ORF63 is able to inhibit apoptosis in these neurons which may aid in the establishment and maintenance of latency.

Interestingly, VZV has been shown to modulate apoptosis in a cell type specific manner. Specifically, VZV induces apoptosis in multiple skin cell types such as MeWo cells (51) and human fibroblasts (HFs) (52) (Figure 2). It was identified in MeWo cells that VZV infection caused a downregulation in Bcl-2 expression, a known anti-apoptotic protein (51). This downregulation of Bcl-2 has also been observed in SVV infection, where apoptosis was induced in infected monkey kidney cells via the intrinsic apoptotic pathway (53). It remains to be determined whether the downregulation of Bcl-2 directly leads to intrinsic apoptosis induction or whether there are other factors involved.

VZV has also been shown to induce apoptosis in immune cells such as T cells, B cells, and monocytes (54–56), however the factors which cause this induction are unclear. Investigating whether the downregulation of Bcl-2 occurs in VZV induced apoptosis in human immune cell types would be pertinent to determine whether VZV apoptosis induction occurs through a similar pathway in all cell types. Overall, it is not clear whether specific VZV gene products cause the induction of apoptosis as a strategy to increase viral dissemination, or rather whether the apoptosis induction is an intrinsic cellular response to limit viral replication and spread.

In contrast to some skin cell types and immune cells, VZV does not induce apoptosis in neurons (Figure 2). This was first identified in the context of primary human sensory ganglionic neurons, whereby VZV could productively infect dissociated human fetal dorsal root ganglia (DRG) cultures, but did not induce apoptosis (52). In intact human fetal ganglia, VZV was also shown to infect neurons without apoptosis induction (57). This phenomenon has been demonstrated in various other neuronal models such as the SCID-hu xenograft DRG mouse model, where explanted human neurons displayed less apoptosis induction than was observed within VZV-infected SCID-hu skin cells (58). VZV also does not induce apoptosis in neurons derived from human neural stem cells (59, 60). Interestingly, in post-mortem ganglia samples from patients with herpes zoster at the time of death, neurons were not identified as being apoptotic, however other cells within the ganglia did display apoptotic markers (61).

### Contribution of VZV ORFs in the Inhibition of Apoptosis

The ability of VZV to protect neurons from apoptosis induction was attributed to ORF 63, using a recombinant virus which was able to express only one copy of the diploid ORF63 gene (62). However, as ORF63 is a potent viral transactivator, it was unclear whether its impact was due to an effect on another VZV ORF. More recently, using lentiviral expression of ORF63 in the differentiated SH-SY5Y neuronal cell line model, it was confirmed that VZV ORF63 could protect against intrinsic apoptosis induction (63). Interestingly, this was also observed in a human keratinocyte cell line known as HaCaTs, suggesting that ORF63 when expressed alone can protect multiple cell types from apoptosis induction (63). VZV infection was also shown not to induce apoptosis in HaCaT cells, a finding which has been previously reported in VZV-infected human papillomavirus (HPV)-immortalized keratinocytes (64). It would be interesting to examine VZV apoptosis induction in the context of primary human keratinocytes, as cell lines can have deficiencies in the apoptotic pathway, which makes them less sensitive to apoptosis induction (65, 66). To date it remains unclear as to why certain cell types are protected from apoptosis during VZV infection and others are not, however there is evidence to suggest that VZV alters the transcriptional profile of apoptotic genes differentially in neuronal cells vs. HFs (67).

Cell type specific modulation of apoptosis is a crucial component of VZV research due to its link to VZV pathogenesis. As VZV establishes life-long latency in neurons of the DRG, the inhibition of apoptosis in neurons is critical for viral maintenance of latency and the establishment of reactivation (68). In contrast, within productive infection in the skin, the induction of apoptosis in HFs may aid in viral dissemination. In the context of VZV ORF63, it will be useful to investigate whether it can protect other cell types when expressed by itself. If this were the case it would suggest that even in productive infection in HFs where apoptosis is induced, the gene product may delay the onset of apoptosis long enough for the virus to replicate. The ORF63 transcript is also one of the major transcripts produced during VZV latency (69), thus it may play a role in apoptosis protection in this context. The mechanism of ORF63 inhibition of apoptosis is still unknown but may be related to its relocalization within the cell during apoptosis induction (63).

Other VZV gene products have also been shown to play a role in apoptosis inhibition. For example, VZV ORF66 inhibits apoptosis in T cells, as evidenced by T cells undergoing apoptosis more readily when infected with a virus in which ORF66 protein expression is impaired (70). Investigation of whether ORF66 can protect against apoptosis when it is expressed alone in immune cells and other cell types would be a potential avenue for future research. VZV ORF12 has been shown to interact with the extracellular-signal-regulated kinases (ERK) signaling pathway in MeWos and fibroblasts (71, 72). This optimizes the capacity for viral replication and causes the inhibition of the apoptosis pathway (71, 72). Specifically, ORF12 enhances the phosphorylation and activation of Akt in a Phosphatidylinositol-4,5-bisphosphate 3-kinase dependent manner (PI3K) (73). This activation was associated with increased levels of cyclin B1, cyclin D3, and the phosphorylation of glycogen synthase 3β (GSK-3β) (73), which are crucial in advancement of the cell-cycle. It has also been reported that the activation of ERK signaling pathway causes the phosphorylation and inhibition of Bim (74). Bim is a pro-apoptotic member of the Bcl-2 family that is usually involved in the propagation of the intrinsic apoptotic pathway (75). Thus, the ability of ORF12 to stimulate cell cycle progression via the ERK signaling pathway can also cause the inhibition of intrinsic apoptosis (74). The effect of ORF12 on apoptosis and cell-cycle progression in neurons is yet to be investigated.

It is clear VZV encodes multiple ORFs with anti-apoptotic mechanisms, demonstrating the importance of modulating apoptosis for viral replication and spread. Interestingly, when expressed alone or deleted from VZV, these genes have an anti-apoptotic effect in cell types where VZV is known to induce apoptosis. It will be important to determine whether these gene products delay the onset of apoptosis in vulnerable cell types during VZV infection as this could be a critical component of VZV pathogenesis in the skin and during reactivation. Furthermore, it would be beneficial to determine whether VZV can protect against other forms of cell death, as when apoptosis is inhibited other cell death forms such as necroptosis can occur to limit viral spread (46). HSV-1 has been shown to inhibit necroptosis (76) and as VZV is closely related to HSV-1, this warrants investigation in the context of VZV.

## Innate Immune Recognition and VZV Interference

The innate immune response to VZV involves the recognition of viral pathogen associated molecular patterns (PAMPs) via PRRs, which triggers inflammatory cytokine secretion and/or cell death. Of the Toll-like receptors (TLRs), Wang and co-workers demonstrated that exposure of monocytes to VZV induced TLR2 and NFκB dependent secretion of interleukin (IL)-6. Furthermore, this report suggested sensing of VZV involved cell-surface TLR2 binding to virion envelope glycoproteins (77). Recently, sensing of VZV through endosomal TLR3, which senses dsRNA has also been proposed (78). The significance of TLR3 sensing initiating anti-VZV responses has been inferred from individuals with defects in genes of the TLR3 pathway suffering from severe varicella resulting in VZV encephalitis (78). Interestingly, there has also been evidence of patients with TLR3 mutations suffering from HSV-1 encephalitis but not VZV encephalitis (79). This may suggest that differing mutations in TLR3 may predispose patients to different susceptibilities to viral infections or that TLR3 sensing is more critical for controlling HSV-1 than VZV. Patients with mutations in downstream components of TLR signaling such as interleukin-1 receptor-associated kinase 4 (IRAK-4) and MyD88 are not susceptible to viral infections such as VZV, highlighting the functional redundancy in the TLR pathogen sensing pathway (80). In particular, it has been shown that IRAK-4 deficient patients can control viral infection through both TLR3 or TLR independent production of type I IFN (81).

TLR3 is known to be expressed in human neurons and peripheral nerve Schwann cells (82, 83), thus implying TLR3 may play a pivotal role in controlling VZV spread in the nervous system. More recently there was a case report describing a 28 year old individual suffering from multiple recurrences of herpes zoster opthalmicus- a disease primarily seen in immunocompromised individuals or elderly individuals following VZV reactivation (84). This study revealed a novel TLR3 variant in an otherwise immunocompetent individual was associated with recurrent herpes zoster opthalmicus. Interestingly, the patient's fibroblasts but not antigen presenting cells (APCs) showed an inability to respond to stimulation with a TLR3 agonist (84). This report further supports the notion of TLR3 in innate activation and control of VZV infection.

Retinoic acid-inducible gene I (RIG-1) is a cytoplasmic PRR which senses both RNA and DNA viruses and can result in the production of the type I IFN response (85). Knockdown of RIG-1 in the context of VZV infection does not affect viral titers in MRC-5 cells, however in human dermal fibroblasts (HDF) RIG-1 overexpression caused a significant suppression of viral replication (86). This suggests that in HDF a RIG-1 induced IFN response may play a role in controlling VZV infection, however RIG-1 is not essential for the control of VZV replication (86). As of yet there have been no VZV ORFs implicated in the inhibition of RIG-1 sensing, however VZV ORFs do target downstream transcription factors such as NFκB, that are involved in the production of inflammatory cytokines (87).

Monocytes and other myeloid cells are also able to sense virus through NLRs, which trigger a pro-inflammatory response through inflammasome formation (88). Interestingly, it has been demonstrated that VZV induces the formation of an inflammasome through the NLR, NLRP3, leading to secretion of pro-inflammatory IL-1β following infection of the monocytic THP-1 cell line (89). Furthermore, in SCID-hu mice with human skin xenografts, NLRP3 was detected throughout VZV infected skin, indicating a function for NLRP3 inflammasomes in local cutaneous immunity (89). The role of NLRP3 inflammasomes and whether VZV can actively modulate this at other key sites of infection such as human ganglia has yet to be explored.

Another intrinsic defense mechanism limiting VZV infection in human skin is the formation of promyelocytic leukemia (PML) cages in infected epidermal cells, which trap VZV nucleocapsids resulting in inhibition of virion assembly (90). Wang and colleagues demonstrated that the ability of VZV ORF61 to bind small ubiquitin-like modifier (SUMO) is required to counterbalance PML nuclear body-mediated control of VZV replication, and enable the formation of skin lesions during varicella and herpes zoster (91). Recently it has been shown that human skin cells including dermal fibroblasts and HaCaT keratinocytes can sense cytosolic VZV DNA through stimulator of interferon genes (STING), triggering secretion of type I and III interferons, which limited VZV replication (86).

## VZV Modulation of the Interferon (IFN) Responses

Interferons (IFNs) are key anti-viral cytokines that mediate their activity through the induction or upregulation of a suite of interferon stimulated genes (ISGs), which have a range of anti-viral activities (92). Recognition of incoming pathogens by both cell-surface and intracellular PRRs initiates a signaling cascade driving the production of type I IFNs through the action of key transcription factors including interferon regulatory factor (IRF) 3 and NF-κB. The IFNs produced can then signal through canonical IFN receptors on the cell-surface leading to activation of a JAK-STAT signaling cascade to drive ISG production (92).

### Clinical Observations Regarding the Importance of IFN in the Control of VZV Infection

Given the key role of IFNs in controlling many viral infections it is unsurprising that IFNs can also profoundly modulate VZV infection. This is emphasized by a number of *in vivo* observations. More than 30 years ago a clinical trial to evaluate the efficacy of IFNα in inhibiting VZV infection in children suffering from cancer indicated that IFN treatment could limit the dissemination of severe varicella lesions (93). Analogously, in the SCID-hu skin model of VZV infection, blocking the type I IFN receptor by neutralizing antibody led to a 10-fold increase in virus titer compared to control antibody treated mice (16).

Patients presenting with primary immunodeficiencies characterized by defects in interferon signaling pathways are also associated with acute VZV infection. Recently four cases of otherwise healthy children presenting with severe VZV infections in both the lungs and CNS were identified as having missense mutations in individual subunits of RNA polymerase III (94). RNA polymerase III acts as a sensor of AT-rich DNA that can drive IFN production (95). Leukocytes isolated from such patients had significantly reduced capacity to transcribe both type I and type III IFNs following stimulation with AT-rich DNA which is a specific characteristic of the VZV, but not other, herpesvirus genomes (94). In a separate study, it was reported that two adult patients suffering from severe VZV infections of the CNS also had mutations in specific RNA polymerase III subunits (96). Cells isolated from such RNA polymerase III deficient patients also demonstrated enhanced susceptibility to VZV infection *in vitro* (94, 96). Other primary immunodeficiencies associated with VZV infection and defects in IFN signaling and/or production include defects in DOCK2 (97), DOCK8 (98), and the IFNγ receptor (99).

Patients with rare genetic defects in downstream components of the type I IFN signaling pathway such as STAT1, TYK2, and NEMO have been shown to increase susceptibility to viral infections such as varicella (100–103). Susceptibility to viral infection has also been reported in patients with mutations in STAT2 (104). STAT2 helps form the ISGF3 complex which binds to IFN sensitive response elements (ISRE) (105). These patients had VZV infection but did not experience severe complications, which questions the importance of type I IFN in controlling VZV infection (104). Interferon independent pathways have also been shown to play a critical role in the control of viral infection and may be able to compensate for the lack of type I IFN response in these patients (106).

### VZV Modulation of IFN Signaling Pathways

The key regulatory role of interferons during VZV infection is underlined by the range of mechanisms encoded by the virus to regulate both the production of and response to IFNs. VZV encodes at least three gene functions that can limit the production of type I IFN with a particular focus on disruption of signaling through IRF3. The serine threonine kinase encoded by the ORF47 gene induces an atypical phosphorylation of IRF3 which inhibits the self-dimerization of IRF3 required for efficient IFNβ induction (107). ORF61 can directly interact with the IRF3 protein promoting IRF3 ubiquitination and subsequent proteasomal degradation (108). The IE62 protein was also demonstrated to block IRF3 phosphorylation at three specific residues on IRF3, inhibiting activation of an IFN stimulated reporter element construct (109). Given the key role of NF-κB in amplifying type I IFN transcription it is likely that the identified role for E3 ubiquitin ligase domain of ORF61 in limiting TNF induced NF-κB activation (87) will also contribute to the inhibitory effect of VZV infection on IFN induction.

More recently, it has been identified that VZV can induce suppressor of cytokine signaling 3 (SOCS3) to modulate type I IFN signaling and viral replication (110). Multiple viruses have been shown to increase SOCS3 expression during infection to suppress signal transduction activated by IFNβ (111). VZV infection of fibroblasts (MRC-5) and macrophages (THP-1) caused an increase in IFNα and IFNβ transcripts in early phases of infection whereas in keratinocytes (HaCaTs) IFNα and IFNβ transcripts persisted until later time-points (110). As these cells were infected at a 1:1 ratio with VZV infected HFFs, it is unclear whether inoculating VZV infected HFFs, could be masking the effect of VZV infection on IFNα and IFNβ transcription in these different cell types. An elevation in SOCS3 protein expression was correlated to a reduction in phosphorylation of STAT3 which is required to drive type I IFN induced gene expression (110). As the effects of mock inoculating HFFs were not addressed in the protein analysis of SOCS3 and pSTAT3, it may be pertinent to perform cell associated infections with the same cell type to exclude effects of using different inoculating cells. Overall, it would be interesting to determine if the induction of SOCS3 by VZV extends to different cell types such as neurons and if so, what is driving the increased expression of SOCS3 in the context of VZV infection. When SOCS3 was knocked down in MRC-5 cells, VZV viral gene expression was inhibited suggesting that the induction of SOCS3 by VZV may be critical for VZV spread and pathogenesis (110).

### VZV Modulation of IFN in Immune Cells

VZV infection can also target type I and II IFN production through direct infection of immune subsets that play a vital role in anti-viral immunity. Plasmacytoid dendritic cells (pDCs) have the capacity to secrete significant amounts of IFNα following appropriate stimulation (112). Work from our laboratory first identified the tropism of VZV for pDCs both *in vivo* and *in vitro*, with VZV infected pDCs significantly inhibited in their capacity to produce IFNα after stimulation with a TLR9 agonist (113). More recently our identification of the pronounced tropism of VZV for primary human NK cells (114) (covered in more detail in section on NK cells and VZV) led to the observation that such cells have a greatly diminished capacity to produce the type II IFN, IFNγ, following stimulation with PMA/ionomycin (115). Given the tropism of VZV for potent immune effector cells it will be intriguing to determine if this inhibition of IFNγ production also extends to other immune cells, such as CD4^+^ T cells, that also have the capacity to produce this key anti-viral cytokine.

VZV also has the capacity to regulate the activity of both type I and type II IFNs through disruption of signaling downstream of IFN receptor binding. Following IFNγ stimulation, STAT1 phosphorylation, a key signaling event in intracellular transduction of IFN, was increased in human tonsillar T cells infected with an ORF66 mutant compared to cells infected with the parental virus (70), implicating this immunomodulatory protein in this response. This mirrors the situation in HFs where IFNγ-induced MHC class II expression was significantly reduced in VZV infected cells through inhibition of STAT1 and Jak2 protein expression (116). Use of the SVV model of VZV infection indicated that SVV can inhibit IFNα and IFNγ induced ISG expression (117, 118) including ISG15 and Mx1 with such phenotypes recapitulated with ectopic expression of SVV ORF63 alone (117). Heterologous expression of the VZV homolog ORF63 in HFs also reduced levels of IRF9 mirroring the simian homolog. STAT2 phosphorylation although reduced during VZV infection was not targeted by ORF63 (117), suggesting additional as of yet unidentified viral gene products are responsible.

Despite the numerous identified mechanisms that VZV employs to regulate the effects of IFN, it is clear that *in vitro* IFNs have the capacity to directly inhibit VZV infection. Comparison of the ability of IFNα and IFNγ to block infection demonstrated that IFNγ has more pronounced effects on VZV replication in human embryonic lung fibroblasts (119). Another recent report indicates there are cell type specific activities in the relative ability of IFNβ and IFNγ to limit virus production. IFNγ could profoundly inhibit VZV production in ARPE-19, A549, MRC-5 but had only very limited capacity to inhibit infection in MeWo cells, where IFNβ retained the capacity to significantly reduce viral yield (120). IFNγ could also promote survival of VZV infected neurons to potentially ensure the efficient establishment of latency (121). More recently, Como and colleagues demonstrated that Type I IFNs had an inhibitory effect on VZV replication and spread in VZV infected human iPSC derived neurons *in vitro* (122). Furthermore, the SCID-hu DRG model revealed VZV infection of DRG resulted in an increase in pro-inflammatory cytokines as well as IFNα and IFNγ (123). Further studies to understand the distinct activities of type I and II IFNs in regulating infection will potentially tease apart the roles played by distinct IFNs in regulating infection during different phases of the viral lifecycle. Similarly the role of type III IFNs in viral infections is becoming clear, particularly at mucosal sites, and a recent report indicated that VZV infection can promote IFNλ1/3 and IFNλ2 production in keratinocytes in a STING dependent manner and IFNλ has direct anti-viral activity *in vitro* (86). Additional study will be required to fully define the role of type III IFN (IFNλ) during VZV infection.

## VZV Infection of Dendritic Cells and Modulation of Immune Functions

DCs are key immune effectors during viral infection as they are professional APCs instrumental in inducing and modulating anti-viral immune responses. DCs are closely implicated during VZV disease as they are present in lymph nodes and other lymphoid tissues significant to VZV pathogenesis, such as tonsils, as well as residing and migrating through the skin (124). DCs sense invading pathogens and induce innate and initiate adaptive immune responses. DCs have the ability to uptake viral proteins, process, and present antigenic peptides loaded onto major histocompatibility complex (MHC) class I and class II molecules that can be subsequently recognized by CD8^+^ and CD4^+^ T cells, respectively. The interaction of DCs and antigen-specific T cells results in T cell activation and culminates in defining the phenotype of T cells, and instructs the overall immune response against a viral pathogen, such as VZV (125). Given the pivotal role DCs play in the innate and adaptive arms of the immune response to viruses, they have been postulated to be a prime target for viruses, seeking to evade and/or delay the host response by disrupting their immune function (126).

### VZV Infection of Human Monocyte Derived Dendritic Cells

There have been a number of studies exploring the interaction between VZV and DCs. Work from our laboratory first identified that VZV could productively infect human monocyte derived dendritic cells (MDDCs) *in vitro* and this led to efficient transmission of virus to T cells (9). These findings supported the hypothesis that DCs may be a major target for VZV infection and facilitate virus transport from the site of VZV entry (mucosal sites) to draining lymph nodes where the virus infects T cells. The importance of T cell tropism and dissemination of virus to the skin was elegantly shown by Ku and co-workers, in which SCID-hu mice with human skin grafts inoculated with VZV infected human T cells, developed VZV skin lesions (16). The importance of the DC/T cell axis during VZV dissemination is further supported from SVV experiments. Ouwendijk and co-workers identified infected DC-like cells in the lungs of African green monkeys infected with a recombinant SVV expressing enhanced green fluorescent protein (SVV-EGFP) virus and during viremia, SVV was observed in memory T cells (28).

VZV infected MDDCs *in vitro* showed no significant decrease in cell viability or evidence of apoptosis (9). These results imply VZV has evolved a strategy to limit or prevent the onset of apoptosis in DCs. As discussed earlier, this may provide a transient advantage to the virus, allowing VZV to successfully disseminate during the first critical days after primary infection. Analogously, others have employed the *in vitro* MDDC infection model to demonstrate that the VZV vaccine strain (V-Oka) and virulent VZV clinical isolates equally infect these immune cells (127). Furthermore, Hu and Cohen utilized viruses unable to express VZV ORF10, ORF32, ORF57, or ORF66 proteins and demonstrated there was no impairment for infection of immature DCs. In contrast, when an ORF47 mutant virus was used to inoculate the MDDCs, there was a reduction in VZV infection, suggesting the ORF, which encodes a serine/threonine protein kinase, was required to promote VZV replication (128). These *in vitro* based MDDC infection studies provided an impetus to study the interaction of VZV with various DC cell subsets *in vivo*.

### VZV Infection of Langerhans Cells and Plasmacytoid Dendritic Cells

In the skin, a major site for VZV disease, it has been demonstrated via immunostaining of VZV infected skin lesions that there is a significantly reduced frequency of Langerhans cells (LCs) (113, 127), extending an earlier case report which examined CD1a expression in VZV-infected skin (129). These observations suggest activation and migration of LCs to draining lymph nodes (113, 127). In stark contrast, infiltration of pDCs and other inflammatory DCs was observed in varicella and herpes zoster skin lesions (113, 127, 130). In our assessment of DC subsets in skin during natural VZV infection we explored whether these cells were infected by immunofluorescence (IFA) staining. We identified sporadic VZV antigen-positive LCs in the epidermis and VZV antigen-positive pDCs in regions of cellular infiltrate in the dermis of VZV infected skin (113). Notably the subcellular localization of VZV antigen staining within these DC subsets was consistent with replicating virus, indicating these cells are productively infected *in vivo* (113). We extended these analyses to demonstrate that pDCs and MUTZ-3-derived LC *in vitro* are permissive to productive VZV infection (113). Furthermore, Gutzeit and colleagues demonstrated that human skin LCs and dermal conventional DCs isolated *ex vivo*, when exposed to a virulent VZV strain or v-OKA, were susceptible to VZV infection (127). Together, these *in vitro* and *in vivo* based reports highlight the permissiveness of a range of DC subsets to VZV. The next key question is whether virus infection of these DC subsets impacts their functionality.

### VZV Modulation of MDDC Function

VZV infection of human DCs has been shown to result in the modulation of cell-surface receptor phenotype and immune functions. Mature MDDCs, like their immature counterparts, are also susceptible to productive VZV infection (10) which results in the selective downregulation of key cell-surface immune molecules such as MHC I, CD80, CD83, and CD86. The cumulative effect is reduced stimulation of allogeneic T cells, thus indicating VZV actively manipulates the functional capacity of DCs by rendering them as inefficient activators of T cells (10). It has been previously reported that VZV ORF66, a protein kinase, has the ability to retain MHC I molecules in the Golgi of infected fibroblasts and MeWo cells (131, 132). However, the viral gene product(s) and molecular mechanism by which VZV modulates cell-surface immune molecule expression on mature MDDCs has yet to be elucidated.

Moreover, VZV has been reported to reduce cell-surface expression of apoptosis receptor Fas on infected immature and mature MDDCs, whereas surface levels of MHC II remain unchanged (128). However, the mechanism of Fas regulation in MDDCs is currently unknown. VZV infected immature MDDCs are unable to upregulate the functionally important immune molecules CD80, CD83, CD86, MHC I, and CCR7, which are essential for DC maturation and induction of an effective anti-viral responses (9). The NFκB pathway largely regulates the expression of these immune molecules. Interestingly, VZV has been shown in human epidermal and MDDCs to directly interfere with the host cell NFκB pathway by sequestering NFκB proteins within the cell cytoplasm (87, 133). Furthermore, the E3 ubiquitin ligase domain of VZV ORF61 was required to modulate this pathway, downstream of triggering receptors TLR3, TLR8, and TLR9 (87). Use of the SVV model indicated that SVV, like VZV, can prevent ubiquitination of IκBα and additionally prevents the phosphorylation of IκBα (134). This study also revealed that in addition to SVV ORF61, SVV is likely to encode additional modulators of NFκB signaling, as an ORF61 deletion virus retained its capacity to prevent IκBα phosphorylation and degradation. Thus, it remains possible that both VZV and SVV encode additional ORFs that afford evasion of NFκB signaling.

### VZV Modulation of pDC Function

VZV infection of pDCs and epidermal cells has been observed to occur in the absence of an increase in the type I cytokine IFNα production (16, 113). This is of particular interest for pDCs, as a distinctive functional characteristic is their potent ability to synthesize IFNα following virus infection. Significantly, VZV infected pDCs remain refractory to IFNα production, even when stimulated with a TLR-9 agonist. In the future, it will be important to further define the mechanistic basis of VZV modulating IFNα production by pDCs and identify any viral gene(s) which encode this function. Additionally, pDC also secrete cytokines and chemokines that stimulate activation of effector cells, including B cells, T cells, NK, NKT cells, and also function to present viral antigen to T lymphocytes (135, 136). Elucidating whether VZV interferes with these other pDC functions during infection will therefore be an important consideration of studies to fully define the functional impact of VZV infection of pDCs.

**Figure 3:**
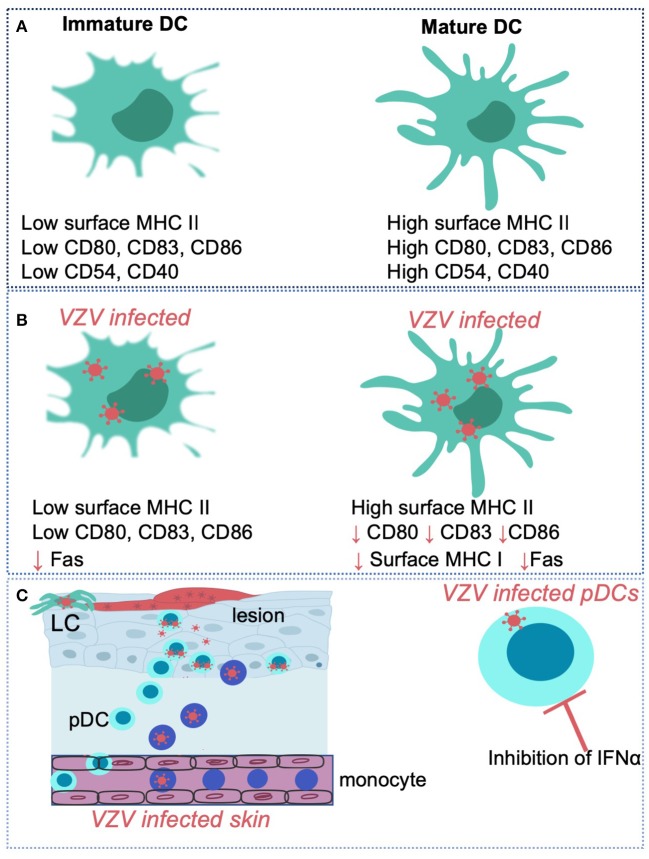
VZV interactions with human dendritic cell subsets and monocytes Immature dendritic cells (DC) are distinguishable from mature DC via differing expression levels of surface markers such as MHC II, CD80, CD83, CD86, CD54, and CD40 **(A)**. VZV has been shown to productively infect human immature and mature monocyte derived dendritic cells and selectively regulate expression of key cell-surface molecules such as CD80, CD83, and CD86 in virus infected cells **(B)**. VZV can also productively infect human Langerhans cells (LCs) an plasmacytoid dendritic cells (pDCs) in the skin **(C)**. VZV infection of pDCs *in vitro* results in the inhibition of IFNα production. VZV also productively infects human monocytes and macrophages in culture.

Interestingly, Gutzeit and colleagues reported the secretion of signature Th1 cytokines (IFNγ and IL-12) was enhanced following infection of MDDCs with (v-OKA) but blocked by a VZV clinical isolate. This impairment of IL-12 secretion was shown to be due to a viral disruption of signaling downstream of TLR2, and proposed to be most likely caused by a VZV glycoprotein within the virion envelope (127). Thus, VZV subversion of the Th1-promoting instruction of human DCs is a novel immune evasion mechanism of clinical VZV isolates. In sum, VZV has encoded a plethora of immune evasion tactics when engaging with various DC subsets (Figure 3). It remains important to further elucidate the molecular mechanisms as well as define the viral proteins directly responsible for these immune evasion strategies. VZV like other herpesvirus family members is likely to encode more than one strategy to manipulate DC functions to provide a transient advantage to the virus.

## VZV Infection and Manipulation of Monocytes and Macrophages

Monocytes and macrophages play a key role in pathogen sensing, immune defense against infection and are important players in resolving inflammation (137). These cells are capable of potent inflammatory and anti-inflammatory responses that define the activation and suppression of a broad range of immune cells (138). There are several different types of macrophages which can be found at various sites within the host and how they respond to different viruses may vary. Given their location in circulation, migratory capacity and tissue-residency, these cell types are highly likely to interact with VZV during the early innate response.

VZV viremia is associated with primary VZV infection and reactivation, and the interaction between VZV and mononuclear cells during these stages of infection has been well-documented (139–141); reviewed in White and Gilden (142). VZV DNA is observable in many mononuclear cell subsets, although few were extensively characterized (19, 143–146). Previously, little focus was drawn on the susceptibility of individual subsets of mononuclear cells to VZV infection, with monocytes and macrophages being no exceptions. Although magnetically isolated CD14^+^ cells from varicella patients harbor detectable copies of VZV ORF62 and VZV gB transcripts (147), original studies exposing primary isolated human monocytes to VZV did not corroborate these findings, suggesting that VZV infection in monocytes was abortive (148, 149). Interestingly, further studies went on to detect VZV gE expression on CD4^−^/CD8^−^ populations of mononuclear cells which were presumed to be monocytes (144). This was subsequently substantiated by a series of reports by Köenig and co-workers, who isolated monocytes from fresh PBMCs and identified VZV gE expression by IFA (54).

More recently however, our laboratory performed an investigation into the susceptibility of human monocytes and macrophages to VZV infection. We reported productive VZV infection of both freshly isolated human monocytes and differentiated macrophages (56). Interestingly, macrophages were highly permissive to VZV infection. This report went on to address the influence of VZV infection of these cell types, indicating that VZV infection influences the antigen presentation potential of monocytes, and predicted that VZV infection substantially impacts monocytes longevity and subsequent ability to generate site-specific macrophages. The failure of VZV infected monocytes to differentiate into monocyte derived macrophages is likely due to reduced viability of infected cells and not the inability of macrophages to support a productive infection. The capacity of VZV to productively infect and modulate the function of monocytes may enhance the ability of VZV to establish an infection in the host.

This work was corroborated by a report demonstrating VZV infection of monocytes, NKT cells and B lymphocytes (150) and by productive infection of a THP-1 monocytic cell line (89). Although evidence suggesting monocyte differentiation to macrophage may be influenced by VZV infection *in vivo*, macrophage infection *in vitro* has previously been observed (56, 148). As such, it is likely that although monocytes and macrophages represent a dynamic axis for the induction and maintenance of anti-viral states, VZV is able to counteract this effective branch of the innate immune system through direct infection and immune evasion strategies.

## NK Cells and VZV: Control and Evasion

NK cells are innate cytotoxic lymphocytes that play a significant role in the immune response against viral infection (151). In peripheral blood, NK cells represent ~5–15% of circulating lymphocytes, while also populating additional key sites for anti-viral immunity such as tonsils, lymph nodes, spleen, lungs, and bone marrow. NK cells can rapidly migrate to sites of inflammation where their activity toward infected cells is mediated by the integration of signals from germline-encoded activating and inhibitory receptors. Activated NK cells will release cytotoxic granules containing perforin and granzymes across the immune synapse, triggering lysis of the infected cell. Additionally, NK cells are potent producers of pro-inflammatory cytokines, such as IFNγ and tumor necrosis factor (TNF).

### Importance of NK Cells in the Control of VZV Infection

The significance of NK cells in the control of VZV infection is particularly apparent in cases of NK cell deficiency. A common motif in individuals with NK cell deficiencies is increased susceptibility to developing severe, often fatal, herpesvirus infections, especially VZV disease (152–157). These case studies indicate that robust NK cell immunity is required for the control of VZV infection. In immunocompetent hosts, several reports have documented increased frequencies of NK cells (158–161), suggesting an active response to infection. Furthermore, in a study of life-threatening varicella cases it was reported that circulating NK cell numbers were significantly lower compared to cases of mild infection, with counts subsequently normalizing during convalescence (160). Recently it has also been demonstrated that NK cells can be rapidly recruited to sites of VZV antigen challenge in previously exposed hosts (162). *In vitro* experiments have also demonstrated that VZV infected cells are sensitive to granulysin (163)–a cytotoxic protein secreted by NK cells as well as cytotoxic T cells. Together, these observations imply a central role for NK cells in the anti-viral immune response to VZV.

While NK cells constitute a key arm of the early innate immune response, VZV can also infect NK cells, potentially using them to disseminate virus (114, 150). During primary infection, the spread of VZV to different sites in the body is considered to be facilitated by the migration of infected T cells (15, 16). This has been supported by clinical observations of immunocompetent patients with varicella, where VZV could be cultured from PBMCs with lymphocyte morphology isolated during the early stages of infection (144, 164). Later reports then sought to confirm VZV infection of T cells and B cells in patients with varicella (146, 147, 165), and extensive studies have since elegantly investigated the role of T cells in VZV infection (166). However, reports identifying T cell and B cell infection overlooked the third major lymphocyte population present in peripheral blood–NK cells. It is likely that the delayed development of the NK cell field in comparison to the fields of T cell and B cell immunology accounts for these earlier studies failing to acknowledge a possible role for NK cells in VZV pathogenesis. Work from our laboratory demonstrated that human NK cells, in particular the CD56^dim^ subset which predominates in blood, are highly permissive to productive infection with both clinical and vaccine strains of VZV (114). Moreover, VZV infected NK cells are capable of transmitting infection to epithelial or fibroblast cells in culture and can upregulate skin-homing chemokine receptors, suggesting a potential role in viral dissemination during pathogenesis (114). Jones and co-workers in a later study also demonstrated VZV infection of PBMC derived NK cells (150). A case report of severe, persistent varicella identified VZV DNA in NK cells, amongst other lymphocyte populations (161), however targeted investigation of NK cell infection in additional varicella patients is needed to corroborate the *in vitro* findings.

### VZV Manipulation of NK Cell Function

**Figure 4:**
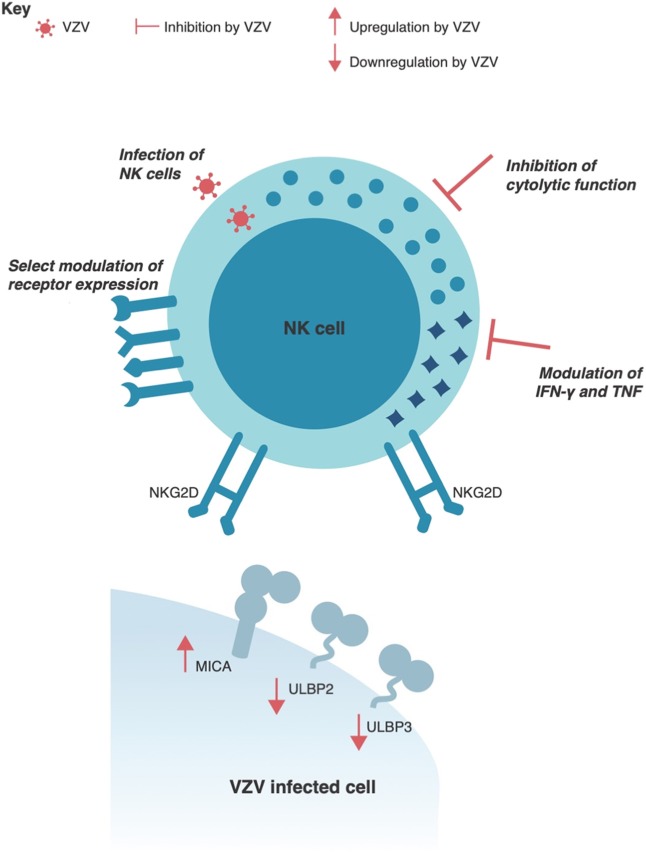
VZV interactions with human Natural Killer cells. VZV has been shown to selectively regulate expression of NKG2D ligands, such as MICA, ULBP2, and ULBP3 in virus infected cells. VZV can also productively infect human NK cells and directly interfere with NK cell function by inhibiting the cytolytic response and modulating IFNγ and TNF cytokine production. Additionally, VZV infection can selectively modulate receptor expression on NK cells.

VZV encodes a number of immune modulatory components to interfere with NK cell detection of infected target cells. Like all other herpesviruses, VZV downregulates the expression of MHC I on the surface of infected cells, which would limit effective CD8^+^ T cell detection of infection (131, 132, 167). However, in response to this common evasion strategy, the immune system counterbalances with NK cell activity which is activated in the absence of cell-surface MHC I. Further modulation of the infected cell-surface is thus required for the virus to reduce detection and clearance by both T cells and NK cells. Specifically, VZV has been shown to reduce cell-surface expression of ULBP2 and ULBP3 (168)–two of eight human ligands detected by the ubiquitously expressed activating NK cell receptor, NKG2D. Interestingly, a third NKG2D ligand, MICA, was found to be upregulated at the total protein level and on the cell-surface of VZV infected cells (168). The differential regulation of NKG2D ligands by VZV is evidence of the dynamic interplay between the virus and NK cell-mediated immune control (Figure 4). Additional evasion of NK cell activity is likely to occur through the downregulation of intracellular adhesion molecule 1 (ICAM-1) (129, 169), which is required for NK and T cell adhesion to target cells to form an immune synapse and clear infected cells. *In vitro* assays have demonstrated that NK cell activity is not enhanced when co-cultured with VZV infected target cells (168), suggesting that VZV sufficiently modulates interactions with NK cells to limit detection and activation. Given the pronounced modulation of these NKG2D ligands and ICAM-1 it will be important for future studies to identity the viral gene products responsible and their mechanisms of action.

In addition to lysing target cells through receptor–ligand interactions, NK cells can also mediate target cell death through antibody-dependent cell-mediated cytotoxicity (ADCC). Expression of CD16 (FcγRIII) on NK cells allows engagement of IgG antibodies bound to a target cell, which typically occurs during anti-pathogen immune responses. VZV infected and bystander NK cells, however, potently downregulate cell-surface expression of CD16 (114), which would hinder ADCC function. Notably, this observation has also recently been documented *in vivo* where CD16 expression was significantly reduced on NK cells that had infiltrated the site of VZV antigen challenge (162). A third mechanism of NK cell cytotoxicity is achieved through Fas–Fas ligand (FasL) interactions which stimulate apoptosis of the Fas-expressing cell. VZV has been shown to reduce cell-surface expression of Fas on infected DCs (128), which would limit NK cell induction of apoptosis in these infected cells. Additionally, VZV infected NK cells themselves have been reported to upregulate expression of programmed death ligand-1 (PD-L1) (150), potentially impeding effective immune responses through the inhibitory signal this transmits. Overall, these alterations to the cell-surface landscape of infected cells are likely to protect VZV from effective immune clearance by NK cells.

Not only does VZV regulate detection of infected cells, we have recently shown that it directly impairs NK cell function (Figure 4). Both infected NK cells and those merely exposed to VZV in co-culture are rendered unresponsive to subsequent target cell stimulation (115). This potent paralysis of NK cell function was found to be dependent on direct contact between NK cells and VZV infected cells. In support of this finding, a report of patients with herpes zoster observed impaired NK cell activity against target cells (158). More recently, decreased serum levels of granulysin has also been reported in varicella patients (170). As the cell count of circulating NK cells was unchanged in these patients, it was suggested that NK cell activity was decreased during varicella, which supports the *in vitro* characterization of inhibited NK cell function by VZV.

Lastly, an important function of NK cells is the secretion of immune modulating cytokines. In relation to the control of VZV, IFNγ, and TNF are readily secreted by NK cells and have strong inhibitory effects on VZV replication (119, 171, 172). These cytokines are also found to be elevated in the serum of varicella patients (173, 174). Despite this, it has been demonstrated *in vitro* that VZV diminishes NK cell secretion of both IFNγ and TNF (115) (Figure 4). This serves as another example of the dichotomy between immune activity necessary for control of VZV and the evasion strategies employed by the virus. As genetically plastic pathogens, viruses only maintain genes of benefit to the survival of the virus, and thus the extent of evasion strategies that subvert NK cell immunity indicates the significance of this cell type in controlling VZV infection. Despite this, our understanding of how VZV interacts with NK cells is only beginning, with many of the most extensive studies on this topic being published in only the last few years. It is likely that we still have much to uncover about the complex interplay between NK cells and VZV.

## Concluding Remarks

VZV has co-evolved with the human host for millions of years (175). In that time there has likely been a dynamic interplay between the emergence of host anti-viral immune responses and subsequently viral mechanisms to evade these defenses. Sensing of viral components and subsequent host cell damage can initiate cell death, the production of type I IFN and pro-inflammatory cytokines to restrict viral spread. VZV produces multiple ORFs such as ORF12, ORF66, and ORF63 to inhibit apoptosis in cells critical for viral dissemination and the establishment of life-long latency. Additionally, VZV can interfere with the type 1 IFN pathway and the production of pro-inflammatory cytokines through the inhibition of pathway components such as IRF3 and NFκB. With the production of pro-inflammatory cytokines and chemokines innate immune cells such as monocytes, macrophages, DCs, and NK cells can target VZV infected cells. VZV has been shown to infect these key immune cells and is able to modulate their function. In this respect, VZV infection modulates expression of key cell-surface immune molecules on DCs, impacts their APC capacity. Furthermore, VZV infection influences the antigen presentation potential of monocytes, and substantially impacts monocytes longevity and ability to generate site-specific macrophages. Recently, VZV was shown to functionally impair NK cells in both their ability to secrete cytokines and lyse virally infected target cells through NK cell dependent cytotoxicity.

There are still many areas of VZV encoded innate immunity manipulation that warrant further investigation. For example, exploring whether VZV protect against other forms of cell death, as when apoptosis is inhibited other cell death forms can occur to limit viral spread. Additional study will be required to fully define the role of type III IFNs during VZV infection. Specifically it will be of interest to understand the distinct activities of type I, II, and III IFNs in regulating infection as this will potentially dissect the roles played by distinct IFNs in regulating infection during different phases of the viral lifecycle. Despite VZV being shown to modulate immune functions of different DC subsets, the molecular mechanisms and VZV proteins directly responsible for these immune evasion strategies has yet to be elucidated. Finally, recent data showing NK cells and other immune cells within PBMC compartment can be infected with VZV provides an avenue to gain a deeper understanding of the impact VZV infection has on immune cell functions and the importance of these cells in viral pathogenesis.

Modulation of the innate immune response ultimately effects the formation and effectiveness of the adaptive immune response. Therefore, it is clear VZV can modulate components of the intrinsic, innate and adaptative immune response to ensure viral dissemination and the establishment of life-long latency. It is critical to dissect the mechanisms of this immunomodulation to provide important insights into VZV pathogenesis which will likely be of benefit when designing new generation vaccines and anti-virals. Furthermore, the study of herpesvirus modulation of immune responses also enhances our general understanding of the complexity of the human immune system.

## Author Contributions

All authors listed have made a substantial, direct and intellectual contribution to the work, and approved it for publication.

### Conflict of Interest

The authors declare that the research was conducted in the absence of any commercial or financial relationships that could be construed as a potential conflict of interest.
